# First-in-human, randomized, double-blind, placebo-controlled, single and multiple ascending doses clinical study to assess the safety, tolerability, and pharmacokinetics of cipargamin administered intravenously in healthy adults

**DOI:** 10.1128/aac.01287-23

**Published:** 2024-07-26

**Authors:** Vinay Kumar Venishetty, Jean Lecot, Amanda Nguyen, Jie Zhang, William T. Prince

**Affiliations:** 1 BioMedical Research, Novartis, Hyderabad, India; 2 Novartis Pharma AG, Basel, Switzerland; 3 BioMedical Research, Novartis, Cambridge, Massachusetts, USA; 4 Novartis Pharmaceuticals Corporation, East Hanover, New Jersey, USA; The Children's Hospital of Philadelphia, Philadelphia, Pennsylvania, USA

**Keywords:** cipargamin, first-in-human, intravenous, malaria, pharmacokinetic, safety

## Abstract

This first-in-human study assessed safety, tolerability, and pharmacokinetics (PK) of cipargamin (intravenous) in healthy adults. It included part 1, single ascending dose [SAD: 10.5 mg–210 mg; *n* = 8 (active: 6, placebo: 2)], and part 2, multiple ascending dose [MAD: 60 and 120 mg daily for 5 days; *n* = 9 (active: 6, placebo: 3)]. Last dose follow-ups were on days 3, 4, and 6 for SAD and 7, 8, and 10 for MAD. Safety and PK review was done at completion of each cohort. We explored the cipargamin use for clinical development in patients with severe malaria. In SAD part, systemic exposure (maximum measured concentration and area under the curve) increased with increasing dose (10.5 mg–210 mg) following single intravenous dose. Cipargamin was eliminated with a mean *T*_1/2_ of 21.9–38.9 h. Volume of distribution (92.9 L–154 L) and clearance (2.43 L/h–4.33 L/h) was moderate and low, respectively, across the dose range. In MAD part, the mean accumulation ratio was 1.51 (60 mg) and 2.43 (120 mg) after once-daily cipargamin administration for 5 days. After day 5, the mean *T*_1/2_ was 35.5 (60 mg) and 31.9 h (120 mg) with twofold dose increase (60–120 mg) resulting in ~2-fold increased exposure. Cipargamin was well tolerated with commonly reported mild gastrointestinal, neurological, and genitourinary events. Increasing exposure to cipargamin showed higher baseline-corrected QTcF, and model-predicted ΔΔQTcF indicated that an effect on ΔΔQTcF ≥10 ms can be excluded up to 6470 ng/mL. However, these results should be interpreted with caution due to inadequate Fridericia’s QT correction.

**CLINICAL TRIALS:**

This study is registered with ClinicalTrials.gov as NCT04321252.

## INTRODUCTION

Despite being a curable and preventable disease, malaria is prevalent in many countries around the world. Globally, ~247 million cases and 619,000 deaths were reported in 2021 (1). The majority of malaria deaths across the globe are due to infection with *Plasmodium falciparum*, which remains an important threat to public health worldwide (2, 3). It accounts for 99.7% of estimated malaria cases in the African region and affects >50% of the population in other parts of the world (4).

The World Health Organization recommends intravenous (IV) or intramuscular administration of artesunate or quinine for the treatment of severe malaria (5). Although the management and treatment of malaria has improved in the past two decades (1), death rates have plateaued in recent years. Moreover, partial resistance to artemisinins has now emerged in Southeast Asia with early reports of foci in other regions (6–8), and no new treatments for severe malaria have emerged.

The preparation and administration of artesunate injection is complicated (9). On the other hand, quinine has lower efficacy than artesunate with potential toxicity issues (10, 11) and must be given by slow infusion due to the risk of cardiovascular side effects (12, 13). Given the challenges associated with the existing treatments, there is a need for novel antimalarial agents for the treatment of severe malaria.

Cipargamin (KAE609), a spiroindolone, represents a new class of potent, fast-acting, schizonticidal antimalarial drugs. Cipargamin acts through a mechanism that disrupts intracellular Na^+^ homeostasis (14, 15). It has been effective in clearing parasites rapidly when given orally in patients (16) for uncomplicated *P. falciparum* (17). It acts against multiple intra-erythrocytic stages of plasmodia, including gametocytes (15). Following once-daily IV bolus administration to rats (3, 10, and 20 mg/kg/day) and dogs (0.5 to 5 mg/kg/day) for 2 weeks, exposure [maximum observed plasma drug concentration after single-dose administration (*C*_max_) and area under curve (AUC)] of cipargamin was approximately dose proportional (within a factor of twofold relative to the dose increment) with moderate accumulation (≤2-fold) of cipargamin after multiple doses in rats and dogs. Safety pharmacology studies show no adverse effects on the central nervous or respiratory systems following oral or IV administration of cipargamin. There was no evidence of QTc prolongation in a dog telemetry study at single IV and oral doses of up to 10 mg/kg. However, in a 2-week IV study in dogs, a minimal increase in QTc interval was seen at 5 and 10 mg/kg/day and a decrease in heart rate at 10 mg/kg/day on day 9 of the study. However, clinical relevance of this is unclear, as no cardiac safety findings were observed in oral studies (18). Cipargamin is being evaluated as an oral treatment for uncomplicated malaria and has demonstrated good efficacy in several clinical studies. Cipargamin, at single doses of 50 to 150 mg or 10 to 50 mg once daily for 3 days, was associated with very rapid parasite clearance, PCR-corrected adequate clinical and parasitological response at 28 days of >65% in adults with uncomplicated *P. falciparum* malaria and exposures of cipargamin increased approximately dose proportional (10 mg–150 mg). Following administration of cipargamin, once-daily dose for 3 days, mean accumulation ratio was found to be 1.4 (17). Due to the rapid clearance of parasites, cipargamin is a suitable candidate for the treatment of severe malaria. One of the common challenges faced in severe malaria is that patients are unable to tolerate or take oral treatment. Therefore, IV administration is considered the most suitable route for the treatment of patients with severe malaria with cipargamin.

This first-in-human study aimed to assess the safety, tolerability, and pharmacokinetics (PK) of single ascending dose (SAD) and multiple ascending dose (MAD) of cipargamin administered intravenously in healthy adults to further support the clinical development of cipargamin in patients with severe malaria.

## RESULTS

### Demographics and baseline characteristics

This study included 57 healthy participants who were randomized to the SAD part (*n* = 39) and MAD part (*n* = 18). Participants were aged 18–55 years, weighed ≥50 kg (body mass index, range: 18.0 kg/m^2^–30.0 kg/m^2^), including both sexes, passed screening assessments, and provided written informed consent as per inclusion criterion.

In the SAD part, all participants in the pooled cipargamin group (*n* = 30) completed the study, whereas one participant from the pooled placebo group discontinued the study. In the MAD part, all participants in the pooled cipargamin (*n* = 12) and pooled placebo group (*n* = 6) completed the study. The majority of participants were female in the pooled placebo MAD group (83.3%), whereas the proportion of males was higher in all the other groups. The demographics and baseline characteristics are presented in Table S1A and B.

### Safety

A total of 57 randomized participants were included in the safety analysis set. In both parts of the study, no deaths were observed. All the adverse events (AEs) were either grade 1 or grade 2 in severity in accordance with common terminology criteria for adverse events (CTCAE; version 5.0). The occurrence of AEs increased with the dose of cipargamin. However, at the last visit, all the AEs were resolved. No AEs led to the discontinuation of the study treatment. One participant in the cipargamin 120 mg SAD group experienced two serious AEs (testicular embryonal carcinoma and testicular malignant teratoma) not related to the study treatment.

In the SAD part, in the pooled cipargamin group, AEs were observed in 11/30 participants (36.7%); 8/30 participants (26.7%) had study treatment-related AEs. The most frequently reported study treatment-related AEs were (in at least two participants) semen discoloration (four participants, 13.3%), headache, dizziness, and nausea (two participants each, 6.7%). AEs occurring in at least two participants among all 30 participants included headache, semen discoloration, dizziness, and nausea. In the SAD pooled placebo group, 5/9 participants (55.6%) had AEs, and 3/9 (33.3%) reported headaches.

In the MAD part, in the pooled cipargamin group, AEs were observed in 10/12 participants (83.3%); 8/12 participants (66.7%) had study treatment-related AEs. The most frequently reported study treatment-related AEs were dizziness (seven participants, 58.3%) and headache (four participants, 33.3%). All the other AEs were reported by one participant each. AEs occurring in at least two participants among all 12 participants included dizziness, headache, diarrhea, and fatigue. In the pooled placebo MAD group, 4/6 participants (66.7%) had AEs, where 2/6 (33.3%) and 1/6 (16.7%) reported diarrhea and headaches, respectively.

Injection-site findings included mild edema and mild pain in both SAD and MAD groups.

One participant in the cipargamin 210 mg SAD group (30 min post-dose on day 1) and one participant in the pooled placebo SAD group (30 min post-dose on day 1) experienced newly occurring post-dose edema at the injection site. One participant in the cipargamin 210 mg SAD group (30 min post-dose on day 1) and one participant in the cipargamin 120 mg MAD group (30 min post-dose on day 5) experienced post-dose pain at the injection site. In the cipargamin 120 mg MAD group, laboratory values outside the normal range were reported as AEs in one participant: increased C-reactive protein, alanine aminotransferase, aspartate aminotransferase, and blood lactate dehydrogenase. All four AEs were grade 1 and resolved in 2 days. No symptoms of liver illness were recorded for this participant. No clinically relevant findings in hematological, urinalysis, and clinical chemistry parameters were observed in this study. One participant in the pooled placebo SAD group experienced an AE of electrocardiogram (ECG) T wave inversion (severity grade 1; on day 1) which was resolved on day 6. In the cipargamin 60 mg MAD group, one participant had an AE of abnormal ECG (severity grade 1; on day 3), which was resolved on day 4.

Higher baseline-corrected QTcF (∆QTcF) was seen with increasing exposure after single or multiple IV doses of cipargamin. The estimated population slope of the concentration-QTc relationship was positive: 0.00097 ms per ng/mL [90% CI: 0.000506, 0.001437]. The model-predicted placebo-corrected ΔQTcF (ΔΔQTcF) at the geometric mean peak concentration showed that an effect on ΔΔQTcF ≥10 ms can be ruled out at a concentration range of up to 6,470 ng/mL. The presence of QT prolongation, according to guidance from Food and Drug Administration/European Medicines Agency, defines a QT prolongation of more than 10 ms as significant. Our analysis indicates that there is no QT prolongation exceeding this threshold, suggesting the absence of a QT effect in this study. However, it is important to note that the validity of our analysis is debatable due to the inadequate Fridericia’s correction. Consequently, no robust conclusion can be drawn from this study. The inappropriate performance of the Fridericia’s correction method necessitates exploring alternative correction methods, such as individual correction. However, these findings should be interpreted with caution as the Fridericia’s QT correction was not effective in adjusting the heart rate effect and no alternative methods of correction could be used in this investigation due to a lack of thorough baseline QT and heart rate measurements.

### Pharmacokinetics

#### Single dose in healthy participants (10.5 mg–210 mg; IV administration)

**Fig 1:**
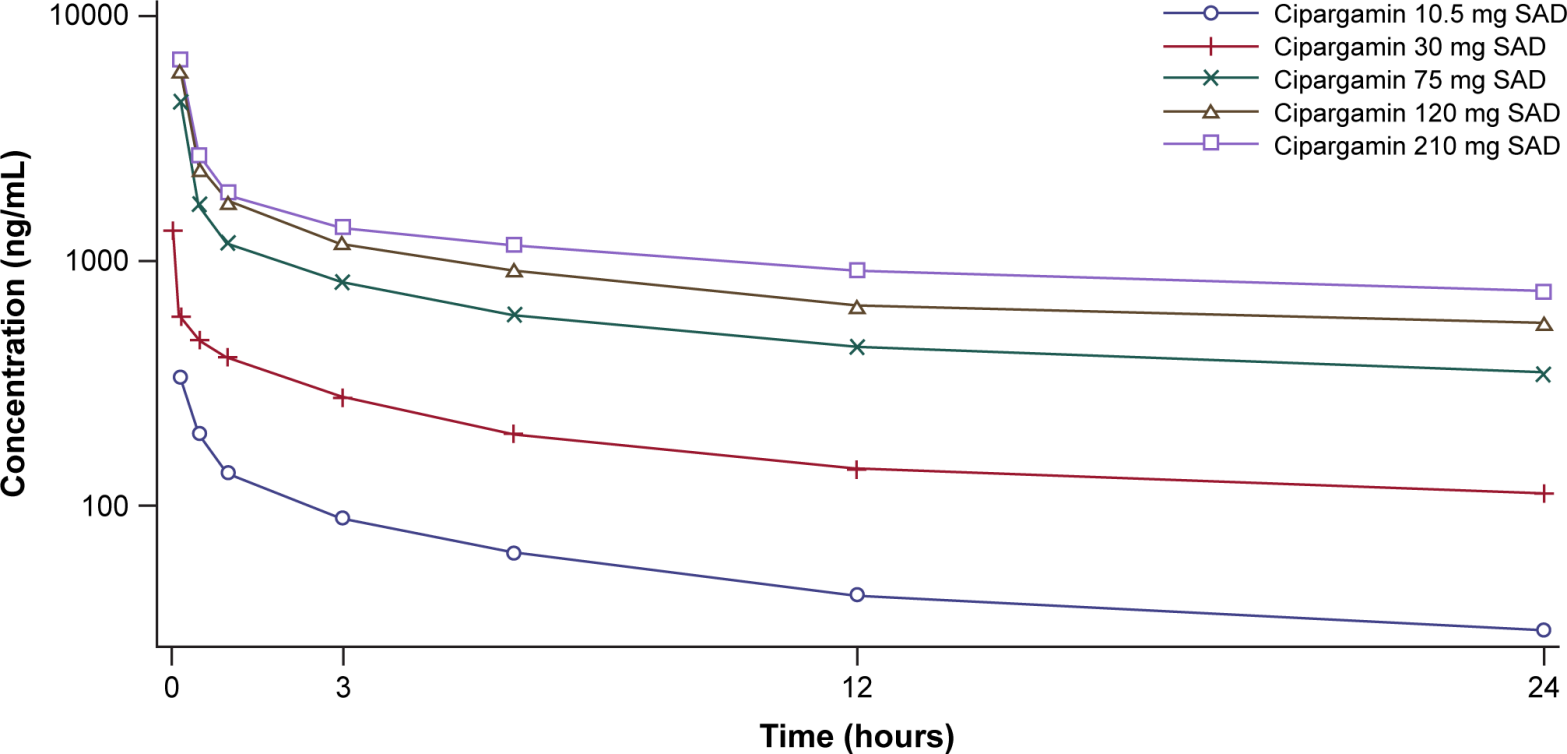
Arithmetic mean (semi-logarithmic) plasma drug concentration-time profiles for cipargamin following IV administration of single ascending doses (SAD part) in healthy adults. Error bars represent ± SD.

The mean plasma drug concentration-time profile of cipargamin following IV administration (10.5 mg–210 mg dose) is shown in Fig. 1. The PK parameters are summarized in Table 1. A trend of increased systemic exposure (*C*_max_ and AUC) was seen with increasing dose after a single IV dose of cipargamin. The mean *C*_max_ ranged from 412 to 6,590 ng/mL over the dose range of 10.5 mg–210 mg. The mean AUC_last_ was in the range of 2.69–60.8 µg-h/mL over the dose range of 10.5 mg–210 mg. Due to the difference in dosing durations between doses <75 mg and ≥75 mg, dose proportionality was not relevant to *C*_max_. AUC_0-∞_ (SAD), AUC_last_ (SAD), and pooled AUC_0-24_ (SAD and MAD) increased in an approximately dose-proportional manner; however, the 90% CIs of the respective slopes did not fall completely within the critical range (0.926, 1.074) to conclude dose proportionality over the whole range; therefore, the statistical assessment of dose proportionality was inconclusive. Following a single IV dosage of 10.5 mg–210 mg, cipargamin demonstrated a moderate volume of distribution (92.9 L–154 L) and low clearance (2.43 L/h–4.33 L/h). Cipargamin was eliminated with a mean *T*_1/2_ of 21.9–38.9 h.

**TABLE 1:** Summary of pharmacokinetic parameters of cipargamin following IV administration of single ascending doses (SAD part)^*a*^^,^^*b*^

Parameter	Cipargamin 10.6 mg SAD *n* = 6	Cipargamin 30 mg SAD *n* = 6	Cipargamin 75 mg SAD *n* = 6	Cipargamin 120 mg SAD *n* = 6	Cipargamin 210 mg SAD *n* = 6
*C*_0_ (ng/mL)	512 ± 144 (28.2)	2,090 ± 1,670 (79.9)	NA	NA	NA
*C*_max_ (ng/mL)	412 ± 101 (24.4)	1,510 ± 1,000 (66.4)	2,910 ± 1,340 (46.0)	5,930 ± 1,190 (20.1)	6,590 ± 1,290 (19.5)
*T*_max_ (h)	0.17 (0.02–0.2)	0.03 (0.03–0.21)	0.33 (0.18–0.55)	0.17 (0.17–0.18)	0.17 (0.17–0.20)
AUC_last_ (h × ng/mL)	2,690 ± 977 (36.3)	9,540 ± 1,640 (17.2)	25,400 ± 1,770 (7.0)	53,700 ± 20,900 (39.0)	60,800 ± 11,600 (19.0)
AUC_0–∞_ (h × ng/mL)	2,770 ± 990 (35.7)	9,750 ± 1,730 (17.8)	25,600 ± 1,730 (6.8)	57,400 ± 22,100 (38.6)	62,000 ± 11,500 (18.6)
AUC_0-24_ (h × ng/mL)	1,450 ± 330 (22.7)	4,540 ± 673 (14.8)	13,500 ± 2,230 (16.5)	23,200 ± 7,920 (34.2)	26,200 ± 3,600 (13.7)
*T*_1/2_ (h)	27.7 ± 8.89 (32.1)	30.5 ± 6.79 (22.3)	21.9 ± 6.76 (30.8)	38.9 ± 12.8 (32.9)	29.4 ± 5.39 (18.3)
CL^*c*^ (mL/h)	4,330 ± 1,890 (43.6)	3,150 ± 507 (16.1)	2,940 ± 192 (6.5)	2,430 ± 1,120 (45.9)	3,500 ± 700 (20.0)
*V*_*Z*_ (mL)	154,000 ± 20,400 (13.3)	138,000 ± 34,000 (24.7)	92,900 ± 29,600 (31.9)	124,000 ± 32,700 (26.4)	151,000 ± 50,400 (33.4)

^*a*^ Statistics are mean ± SD (CV%) except *T*_max_, for which median (min−max) is provided. Duration of injection was 10 min and 2 min for ≥75 mg and <75 mg doses, respectively.

^*b*^ CV, coefficient of variation; NA, not applicable due to IV infusion.

^*c*^ CL, clearance mL/h.

#### Multiple doses in healthy participants (60 mg once daily and 120 mg once daily for 5 days; IV administration)

**Fig 2:**
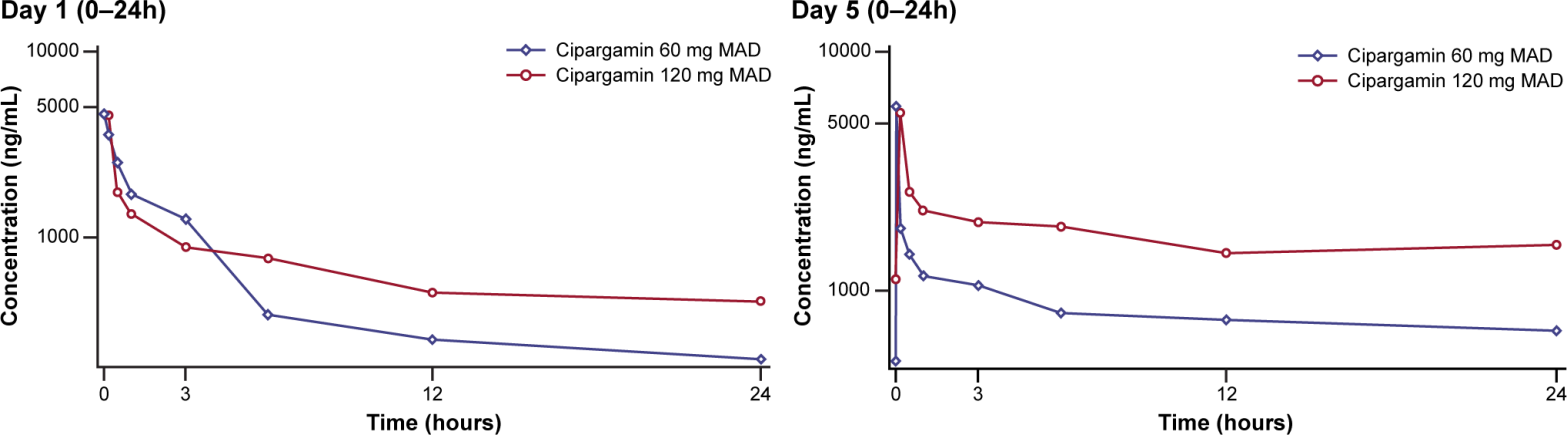
Arithmetic mean (semi-logarithmic) plasma drug concentration-time profiles for cipargamin following IV administration of multiple ascending doses (MAD part) in healthy adults. Error bars represent ± SD.

Day 1 and day 5 mean plasma drug concentration-time profiles of individual dose levels (60 mg once daily for 5 days and 120 mg once daily for 5 days) following IV administration of cipargamin are shown in Fig. 2. The PK parameters are summarized in Table 2. Following a 60 mg once-daily dose regimen, the mean trough concentrations on days 2, 3, 4, and 5 were 221 ± 49.4, 386 ± 95.3, 512 ± 180, and 506 ± 166 ng/mL, respectively. Following a 120 mg once-daily dose regimen, the corresponding trough concentrations were 453 ± 133, 700 ± 173, 1,010 ± 343, and 1,110 ± 440 ng/mL, respectively. Based on trough concentrations, cipargamin is expected to reach steady state after three IV doses. Following 5 days of treatment, a twofold increase in dose (60 g–120 mg) resulted in an approximately twofold increase in exposure. The elimination of half-life (*T*_1/2_) was not calculated on day 1 because of daily dosing of cipargamin. The mean *T*_1/2_ following day 5 dose for the cipargamin 60 mg and 120 mg MAD groups was 35.5 and 31.9 h, respectively. The mean accumulation ratios for the cipargamin 60 mg and 120 mg MAD groups were 1.51 and 2.43, respectively, following once-daily administration for 5 days.

**TABLE 2:** Summary of pharmacokinetic parameters of cipargamin following IV administration of multiple ascending doses (MAD part)^*a*^^,^^*b*^

Parameter	Cipargamin 60 mg MAD *n* = 6	Cipargamin 120 mg MAD *n* = 6
Day 1		
*C*_*0*_ (ng/mL)	4,660 ± 933 (20.0)	NA
*C*_max_ (ng/mL)	3,920 ± 999 (25.5)	4,530 ± 705 (15.6)
*T*_max_ (h)	0.10 (0.03–0.50)	0.20 (0.17–0.18)
AUC_0-24_ (h × ng/mL)	13,200 ± 1,770 (13.4)	16,500 ± 2,290 (13.9)
Day 5		
*C*_*0*_ (ng/mL)	6,360 ± 4,340 (68.2)	NA
*C*_max_ (ng/mL)	4,630 ± 2,670 (57.6)	5,540 ± 2,090 (37.8)
*T*_max_ (h)	0.03 (0.03–0.17)	0.17 (0.17–0.17)
AUC_0-24_ (h × ng/mL)	20,000 ± 4,890 (24.4)	40,600 ± 11,400 (28.0)
AUC_last_ (h × ng/mL)	58,500 ± 23,000 (39.3)	121,000 ± 56,100 (46.5)
*T*_1/2_ (h)	35.5 ± 8.58 (24.2)	31.9 ± 12.5 (39.2)
CL^*c*^ (mL/h)	3,140 ± 717 (22.9)	3,160 ± 907 (28.7)
*V*_ss_ (mL)	175,000 ± 14,700 (8.4)	173,000 ± 29,700 (17.2)
Accumulation ratio	1.51 ± 0.223 (14.8)	2.43 ± 0.393 (16.2)

^*a*^ Statistics are mean ± SD (CV%) except *T*_max_, for which median (min−max) is provided. Duration of injection was 10 min and 2 min for ≥75 mg and <75 mg doses, respectively.

^*b*^ CV, coefficient of variation; NA, not applicable due to IV infusion.

^*c*^ CL, clearance, mL/h.

## DISCUSSION

The findings from this first-in-human-study demonstrated that cipargamin (IV) was well tolerated in healthy adults in both single (SAD: 10.5, 30, 75, 120, or 210 mg or placebo) and multiple ascending doses (MAD: 60 mg q.d. for 5 days, and 120 mg q.d. for 5 days) with occurrence of diarrhea, nausea, headache, dizziness, and semen discoloration. The profiles and dose relatedness of AEs in this study were similar to the results from the study with single (1 mg–300 mg) and multiple (10 mg–150 mg daily for 3 days) oral doses of cipargamin investigated following a very similar design (19). These AEs were generally mild in severity and not expected to have a significant impact on the safety of the participant. Semen discoloration was caused by a yellow-colored metabolite of cipargamin, M23, which was the main metabolite from oral studies. M23 has no cytotoxic potential against a human hepatic cell line; no azoospermia, diminished spermatozoon motility, or other clinically significant abnormalities were recorded in patient samples (19, 20).

Another important parameter, QT interval, can be affected by malaria and is associated with severity of the disease. It has been observed that the marked QT interval shortening and increased sensitivity to changes in heart rate is greater in severe versus uncomplicated malaria. A similar observation is true for *P. falciparum* versus *Plasmodium vivax* infection (14, 20). The preclinical studies in cipargamin have suggested a potential risk for QTc prolongation following IV administration (21). In this present study, higher baseline-corrected QTcF was observed with increasing exposure following single or multiple IV doses of cipargamin. The maximum prolongation seen was 6.3 msec and the results suggest that a change of more than 10 msec can be excluded up to the maximum concentrations studied. Two subjects (one placebo-treated subject in the SAD part and one cipargamin-treated subject in the MAD part) experienced AEs associated with abnormal ECG. Both events were of grade 1 severity and reversible. A QTcF increase >30 ms to ≤60 ms was observed in two KAE-treated subjects in the MAD part, and no relevant AEs were reported. This study was not designed to measure QTcF changes. However, such results should be interpreted with caution as the Fridericia’s QT correction was not effective at appropriately adjusting the heart rate effect and no alternative methods of correction could be applied due to lack of intensive baseline QT and heart rate measurements in this study. Possible effects on QTcF should be explored further in future studies. None of the participants discontinued from study treatment due to AEs. Overall, there were no findings in this study that would have a significant negative impact on its benefit-risk profile, considering cipargamin is being developed to treat severe malaria.

The results demonstrated a PK approximate-dose proportionality. Systemic exposure in terms of AUC_inf_, AUC_last_, and AUC_0-24_ increased in an approximate dose-proportional manner in the single-dose cohorts. However, statistical assessment of dose proportionality was inconclusive as the 90% CIs of the respective slopes did not fall completely inside the critical range. In the single-dose cohort, elimination of half-life (*T*_1/2_) was in the range of 21.9–38.9 h. Administration of multiple doses showed an accumulation ratio in the range of 1.51–2.43 and the *T*_1/2_ was 31.9–35.5 h. Based on trough concentrations, cipargamin is expected to reach steady state after three IV doses. After 5 days of treatment, twofold increase in dose (60 mg–120 mg) resulted in about twofold increase in exposure.

There were no findings in the study that would exclude administering this compound in patients with severe malaria. The overall findings from the study indicate that cipargamin (IV) was well tolerated at single doses of up to and including 210 mg and at 120 mg daily for 3 days. Further investigations in clinical studies to develop it for the treatment of severe malaria are warranted.

## MATERIALS AND METHODS

### Study design

This was a randomized, double-blind, placebo-controlled, single and multiple ascending IV dose study in healthy participants. It was conducted at a single center in Belgium from 22 July 2020 to 10 November 2020. The study comprised two parts. Part 1 involved five SAD cohorts. In each SAD cohort, eight participants were randomized (3:1) to receive different IV dose levels of cipargamin (10.5, 30, 75, 120, and 210 mg) or placebo. The placebo was indistinguishable from cipargamin solutions. The SAD part had a screening period of a maximum of 28 days, a baseline period from day −1 day to dosing, a treatment period (day 1), and a follow-up period (days 2–6).

**Fig 3:**
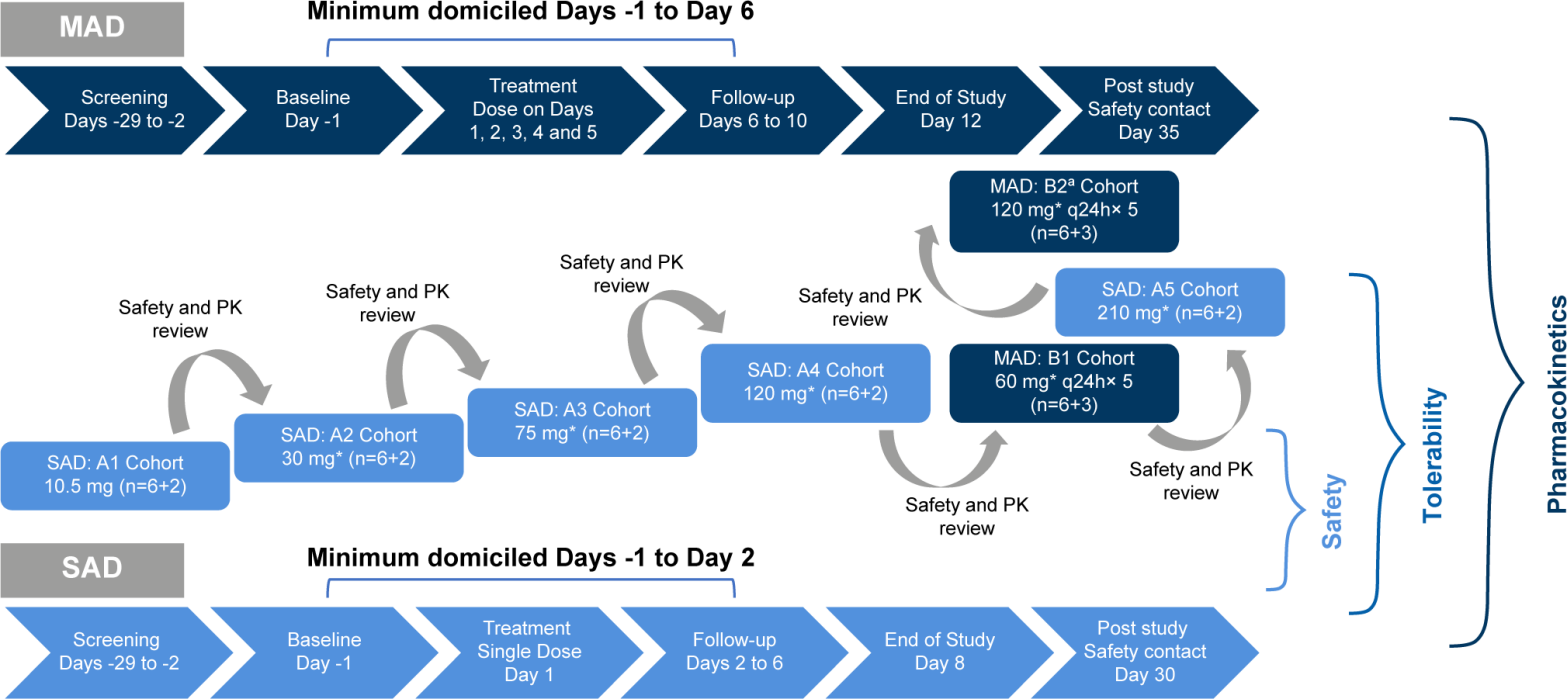
Study design—SAD and MAD. *Dose levels are provisional and might change on emerging data on the study. ^a^Updated cohort B2. q24h, every 24 h.

Part 2 involved two MAD cohorts. In each MAD cohort, nine participants were randomized (2:1) to receive successively higher multiple dose levels of cipargamin (60 mg once daily for 5 days, 120 mg once daily for 5 days) or placebo. It had a screening period of a maximum of 28 days, a baseline period from day −1 to dosing, a treatment period (days 1–5), and a follow-up period (days 6–10). In both the parts, after end of study (EOS) visit (SAD EOS day 8, MAD EOS day 12), participants had a post-study follow-up call approximately 30 days after the treatment. Safety and PK parameters were evaluated during domicile period in both the parts. A schematic diagram of the study design of two dose cohorts is shown in Fig. 3.

Single or multiple IV cipargamin (15 mg/mL) or placebo doses were administered via IV bolus over ~2 min (<75 mg) or IV infusion over ~10 min (≥75 mg).

None of the participants were impacted by coronavirus disease 2019 (COVID-19) during the study. Hence, COVID-19 had no impact on the study conduct, planned analysis, the integrity, or interpretability of the study results.

### Study participants

Participants with good health as determined by past medical history, physical examination, and laboratory tests at screening were included. Male participants were required to comply with barrier contraceptive methods throughout the study and up to ≥2 weeks following the last dose. Female participants with no child-bearing potential, who were not nursing, and with a negative pregnancy test result at screening and baseline were included. Only medications required to treat adverse events were allowed; no medication (including vitamins or herbal remedy) other than study treatment was allowed from the date of signing the informed consent until the post-study safety follow-up call. Participants who had used investigational drugs within five half-lives of screening or 30 days of dosing (whichever was longer), had a history of hypersensitivity to the study treatments, excipients, or drugs of similar chemical class, had significant illness that had not resolved within 2 weeks of the first dose, and were pregnant were excluded from the study. Participants were tested for drugs of abuse, alcohol, and smoking, and if they tested positive, they were excluded from the study.

### Safety assessment

Safety was evaluated by assessing AEs and physical examinations, monitoring body temperature, height, weight, blood pressure, pulse rate, Holter monitoring, ECG, laboratory parameters (hematology, chemistry, urinalysis), and observation of injection/infusion site. AEs were graded using CTCAE version 5.0.

### Pharmacokinetic parameters and assessment

PK samples were obtained from all participants, but only samples from cipargamin-treated participants were analyzed. For bioanalysis, plasma sample preparation consisted of the following steps: protein precipitation, evaporation of the supernatants, and analysis of the reconstituted sample using reverse-phase liquid chromatography-tandem mass spectrometry with electrospray ionization technique. cipargamin [M + 6] was used as the internal standard (please refer to the detailed methodology and sample preparation steps in the supplementary material). The lower and upper limits of quantification were 1.00 ng/mL and 1,000 ng/mL, respectively, using 50 µL/mL of plasma. Cipargamin is stable in human plasma for at least 24 h at room temperature, after three freeze-thaw cycles, and at −75°C±10°C for at least 395 days. The coefficient of variation (CV%; precision) and percent bias (accuracy) for the multiple runs during the period of analysis for low (3 ng/mL), mid (300 ng/mL), and high (750 ng/mL) quality control samples were 11.25, 8.14, and 9.08, and −1.00, 2.00, and −6.27, respectively. PK parameters were evaluated by using the actual recorded sampling times and non-compartmental method(s) with Phoenix WinNonlin (version 8.0). The following PK parameters were determined in this study: AUC from time zero to time t (AUC_0-t_), AUC from time zero to the last measurable concentration sampling time (AUC_last_), AUC from time zero to infinity (AUC_0-∞_), AUC calculated at the end of dosing interval at steady state (AUC_tau_), maximum observed concentration (*C*_max_), elimination of half-life (T_½_), time to reach maximum concentration (*T*_max_), total body clearance of drug from the plasma (CL), apparent volume of distribution during the terminal elimination phase (*V*_*z*_), and apparent volume of distribution at steady state from an IV administered dose (*V*_ss_).

### Statistical methods

The safety analysis set was used for all safety analyses. Descriptive statistics was used for AEs, local tolerance at infusion site, vital signs, ECG, and clinical laboratory evaluations. For Holter ECG data analysis, linear mixed effects modeling was used to explore the relationship between change from baseline in time-matched QTcF and cipargamin plasma concentration. The placebo-corrected change from baseline in QTcF and the two-sided 90% CI were extracted from the model at the geometric mean maximum plasma concentration for each dose group. All PK parameters’ calculations were based on the actual time of sample collection. PK parameters were summarized using arithmetic mean, standard deviation, and CV%, except for *T*_max_ for which median values and ranges were given. The assessment of dose proportionality was performed using a linear regression model. The log-transformed PK parameters (AUC) were the dependent variable with the log-transformed dose as the fixed effect (power model). An estimate of the slope together with 90% CI was provided and compared to the acceptance region for dose proportionality (22).

## Data Availability

The data sets generated during and/or analyzed during the current study are available from the corresponding author on reasonable request.

## SUPPLEMENTAL MATERIAL

Supplemental material aac.01287-23-s0001.docx Supplemental methods; Tables S1 to S3.

ASM does not own the copyrights to Supplemental Material that may be linked to, or accessed through, an article. The authors have granted ASM a non-exclusive, world-wide license to publish the Supplemental Material files. Please contact the corresponding author directly for reuse.
