# Supplementary material for: First-in-human, randomized, double-blind, placebo-controlled, single and multiple ascending doses clinical study to assess the safety, tolerability, and pharmacokinetics of cipargamin administered intravenously in healthy adults
Source: Antimicrob Agents Chemother. 2024 Jul 26;68(9):e01287-23. doi: 10.1128/aac.01287-23 (PMC11373227; doi:10.1128/aac.01287-23)
Supplement: Supplemental material — Supplemental methods; Tables S1 to S3. [file aac.01287-23-s0001.docx]

**Supplementary material**

**Liquid chromatography-tandem mass spectrometry (LC-MS/MS) method for pharmacokinetic sample:**

***Materials and Methods***

Cipargamin (purity 99.1%) and internal standard ^13^C_6_ cipargamin (purity 97.7%) were supplied by Novartis Pharma AG (Basel, Switzerland).

Chromatographic separation was performed using Shimadzu LC-20AD XR Nexera pumps, column oven CTO-20A, and SIL-20AC XR Nexera or SIL-30AC autosampler (Shimadzu, Columbia, MD, USA) on an Ascentis Express C8, 50 x 2.1 mm analytical column (2.7 μm, 2.1 × 50 mm, Supelco, USA) at 50°C. The mobile phases were (A) water containing 0.1% formic acid and (B) acetonitrile containing 0.1% formic acid. The gradient elution conditions were 0.0 – 0.5 min at 30% B, 0.50 – 2.00 min at 30% – 40% B, 2.00 – 2.50 min at 40% B, 2.50 – 2.51 min at 80% B, 2.51 – 3.50 min at 80% B, 3.50 – 3.51 min at 30% B. The mobile phase flow was 0.4 mL/min, and the total run time was 5 min per injection.

A Sciex API4000 mass spectrometer (AB Sciex, MA, USA) coupled with an Turbo IonSpray ionization ion source was used to monitor the mass transitions. The system was operated in positive mode and multiple reaction monitoring (MRM) with optimized parameters as follows: Ion spray voltage 2000 V; source temperature 600°C; Curtain gas 20 units; GS1 (nebulizing gas, N2) 50 units; GS2 60 units. Scan time was 200 ms for both analytes and the internal standards. The MRM mass transitions monitored for cipargamin and its internal standard were m/z 390.1/347.0 and m/z 396.0/353.0, respectively.

***Sample preparation***

The human plasma sample preparation was processed by protein precipitation. A 50 µL volume of plasma (blank, zero, standards, QCs and unknowns) was added to a 1.4 mL polypropylene tube, followed by addition of a 20 µL aliquot of the IS working solution at 150 ng/mL except for the control blanks, to which a 20 µL aliquot of methanol/water (50/50, v/v) was added, and then 250 µL of acetonitrile containing 0.1% of formic acid. This was followed by vortex-mixing for a few seconds. The tubes were centrifuged at approximately 3500 rpm (2400 x g) for about 10 minutes at approximately 5ºC. A 50 µL volume of the supernatants was mixed with 150 µL of water containing 0.1% of formic acid. A 5 µL volume of the solution was injected into LC-MS/MS system.

The lower limit of quantification (LLOQ) was 1.00 pg/mL in plasma for cipargamin, whereas the upper limit of quantification (ULOQ) was 1000 ng/mL. The overall variability of the plasma calibrations standards of cipargamin were 6.6 (% coefficient of variance, [CV]) and −4.7 to 5.0 (% bias), and the overall variability of the plasma quality control samples were 11.5 (% CV) and −6.0 to 4.0 (% bias).

**Table S1.A.** **Demographics and baseline characteristics of single ascending dose (SAD Part) cohort**

| **Characteristics** | **Cipargamin 10.5 mg SAD** | **Cipargamin**  **30 mg SAD** | **Cipargamin**  **75 mg SAD** | **Cipargamin**  **120 mg SAD** | **Cipargamin**  **210 mg SAD** | **Pooled**  **cipargamin SAD** | **Pooled**  **Placebo SAD** | **Total** |
| --- | --- | --- | --- | --- | --- | --- | --- | --- |
|  | **N=6** | **N=6** | **N=6** | **N=6** | **N=6** | **N=30** | **N=9** | **N=39** |
| Age (years)  Mean (SD)  Median (Min – Max) | 49.0 (7.07)  51.5 (35 – 54) | 41.2 (9.89)  42.5 (27 – 51) | 40.5 (13.95)  43.0 (22 – 54) | 41.3 (13.89)  44.0 (22 -54) | 36.2 (6.34)  36.0 (26 – 44) | 41.6 (10.82)  42.5 (22 – 54) | 44.7 (8.83)  43.0 (31 – 55) | 42.3 (10.36)  43.0 (22 – 55) |
| Sex, n (%)  Male  Female | 5 (83.3)  1 (16.7) | 4 (66.7)  2 (33.3) | 5 (83.3)  1 (16.7) | 5 (83.3)  1 (16.7) | 5 (83.3)  1 (16.7) | 24 (80.0)  6 (20.0) | 7 (77.8)  2 (22.2) | 31 (79.5)  8 (20.5) |
| Race, n (%)  White | 6 (100) | 6 (100) | 6 (100) | 6 (100) | 6 (100) | 30 (100) | 9 (100) | 39 (100) |
| Ethnicity, n (%)  Not  Hispanic/Latino | 6 (100) | 6 (100) | 6 (100) | 6 (100) | 6 (100) | 30 (100) | 9 (100) | 39 (100) |
| BMI (kg/m^2^)  Mean (SD)  Median (Min – Max) | 25.2 (1.08)  24.90  (23.8 – 26.8) | 23.9 (3.76)  25.25  (17.4 – 28.0) | 23.5 (2.63)  23.35  (20.5 – 27.4) | 26.1 (2.78)  26.45  (21.8 – 29.3) | 25.0 (1.20)  25.35  (23.0 – 26.2) | 24.7 (2.52)  25.10  (17.4 – 29.3) | 26.7 (2.54)  27.10  (20.7 – 30.0) | 25.2 (2.63)  25.60  (17.4 – 30.0) |
| BMI, Body mass index; SAD, single ascending dose; SD, standard deviation. | | | | | | | | |

**Table S1. B.** **Demographics and baseline characteristics of multiple ascending dose (MAD part) cohort**

| **Characteristics** | **Cipargamin 60 mg MAD** | **Cipargamin 120 mg MAD** | **Pooled cipargamin MAD** | **Pooled placebo MAD** | **Total** |
| --- | --- | --- | --- | --- | --- |
|  | **N=6** | **N=6** | **N=6** | **N=6** | **N=18** |
| Age (years)  Mean (SD)  Median (Min – Max) | 41.8 (8.73)  43.5 (26 – 52) | 38.0 (12.68)  37.5 (19 – 53) | 39.9 (10.57)  42.0 (19 – 53) | 43.2 (12.50)  45.5 (22 – 54) | 41.0 (10.99)  42.0 (19 – 54) |
| Sex, n (%)  Male  Female | 5 (83.3)  1 (16.7) | 4 (66.7)  2 (33.3) | 9 (75.0)  3 (25.0) | 1 (16.7)  5 (83.3) | 10 (55.6)  8 (44.4) |
| Race, n (%)  White | 6 (100) | 6 (100) | 12 (100) | 6 (100) | 18 (100) |
| Ethnicity, n (%)  Not  Hispanic/Latino | 6 (100) | 6 (100) | 12 (100) | 6 (100) | 18 (100) |
| BMI (kg/m^2^)  Mean (SD)  Median (Min – Max) | 24.3 (2.82)  23.80 (21.4 – 28.8) | 25.8 (1.53)  25.75 (24.2 – 28.2) | 25.0 (2.32)  24.80 (21.4 – 28.8) | 25.8 (2.02)  26.00 (23.4 – 28.5) | 25.3 (2.19)  25.25 (21.4 – 28.8) |
| BMI, body mass index; MAD, multiple ascending dose; SD, standard deviation. | | | | | |

**Table S2. A. Overall incidences of adverse events of following IV administration of single ascending doses (SAD part) in healthy adults**

| **Adverse events (AEs)** | **Cipargamin**  **10.5 mg SAD** | **Cipargamin 30 mg SAD** | **Cipargamin**  **75 mg SAD** | **Cipargamin 120 mg SAD** | **Cipargamin 210 mg SAD** | **Pooled**  **cipargamin SAD** | **Pooled**  **placebo SAD** | **Total** |
| --- | --- | --- | --- | --- | --- | --- | --- | --- |
|  | **N=6**  **nE, nS (%)** | **N=6**  **nE, nS (%)** | **N=6**  **nE, nS (%)** | **N=6**  **nE, nS (%)** | **N=6**  **nE, nS (%)** | **N=30**  **nE, nS (%)** | **N=9**  **nE, nS (%)** | **N=39**  **nE, nS (%)** |
| AEs | 0 | 1, 1 (16.7) | 1, 1 (16.7) | 5, 3 (50.0) | 15, 6 (100.0) | 22, 11 (36.7) | 6, 5 (55.6) | 28, 16(41.0) |
| Grade 1 | 0 | 1, 1 (16.7) | 1, 1 (16.7) | 3, 2 (33.3) | 14, 6 (100.0) | 19, 10 (33.3) | 5, 4 (44.4) | 24, 14 (35.9) |
| Grade 2 | 0 | 0 | 0 | 2, 1 (16.7) | 1, 1 (16.7) | 3, 2 (6.7) | 1, 1 (11.1) | 4, 3 (7.7) |
| Grade ≥3 | 0 | 0 | 0 | 0 | 0 | 0 | 0 | 0 |
| Study treatment-related AEs | 0 | 0 | 1, 1 (16.7) | 1, 1 (16.7) | 10, 6 (100.0) | 12, 8 (26.7) | 4, 3 (33.3) | 16, 11 (28.2) |
| Serious AEs | 0 | 0 | 0 | 2, 1 (16.7) | 0 | 2, 1 (3.3) | 0 | 2, 1 (2.6) |
| AEs leading to discontinuation of study treatment | 0 | 0 | 0 | 0 | 0 | 0 | 0 | 0 |
| Treatment-related AEs leading  to discontinuation of study treatment | 0 | 0 | 0 | 0 | 0 | 0 | 0 | 0 |
| AE, adverse event; IV, intravenous; nE, number of AEs in the category; nS, number of subjects with at least one AE in the category (% is based on the number of subjects); SAD, single ascending dose. | | | | | | | | |

**Table S2. B. Overall incidences of adverse events of following IV administration of multiple ascending doses (MAD part) in healthy adults**

| **Adverse events** | **Cipargamin**  **60 mg MAD** | **Cipargamin**  **120 mg MAD** | **Pooled cipargamin MAD** | **Pooled**  **placebo MAD** | **Total** |
| --- | --- | --- | --- | --- | --- |
|  | **N=6**  **nE, nS (%)** | **N=6**  **nE, nS (%)** | **N=12**  **nE, nS (%)** | **N=6**  **nE, nS (%)** | **N=18**  **nE, nS (%)** |
| AEs | 11, 4 (66.7) | 25, 6 (100.0) | 36, 10 (83.3) | 8, 4 (66.7) | 44, 14 (77.8) |
| Grade 1 | 11, 4 (66.7) | 23, 6 (100.0) | 34, 10 (83.3) | 7, 4 (66.7) | 41, 14 (77.8) |
| Grade 2 | 0 | 2, 2 (33.3) | 2, 2 (16.7) | 1, 1 (16.7) | 3, 3 (16.7) |
| Grade ≥3 | 0 | 0 | 0 | 0 | 0 |
| Study treatment-related AEs | 9, 4 (66.7) | 10, 4 (66.7) | 19, 8 (66.7) | 2, 2 (33.3) | 21, 10 (55.6) |
| Serious AEs | 0 | 0 | 0 | 0 | 0 |
| AEs leading to discontinuation of study treatment | 0 | 0 | 0 | 0 | 0 |
| Treatment-related AEs leading to discontinuation of study treatment | 0 | 0 | 0 | 0 | 0 |
| AE, adverse event; IV, intravenous; MAD, multiple ascending doses; nE, number of AEs in the category; nS, number of subjects with at least one AE in the category (% is based on the number of subjects). | | | | | |

**Table S3. A. Incidences of adverse events of following IV administration of single ascending doses (SAD part) in healthy adults**

| **Adverse events (AEs)** | **Cipargamin**  **10.5 mg SAD** | **Cipargamin 30 mg SAD** | **Cipargamin**  **75 mg SAD** | **Cipargamin 120 mg SAD** | **Cipargamin 210 mg SAD** | **Pooled**  **cipargamin SAD** | **Pooled**  **placebo SAD** | **Total** |
| --- | --- | --- | --- | --- | --- | --- | --- | --- |
|  | **N=6**  **n (%)** | **N=6**  **n (%)** | **N=6**  **n (%)** | **N=6**  **n (%)** | **N=6**  **n (%)** | **N=30**  **n (%)** | **N=9**  **n (%)** | **N=39**  **n (%)** |
| Number of subjects with at least one AE^a^ | 0 | 1 (16.7) | 1 (16.7) | 3 (50) | 6 (100) | 11 (36.7) | 5 (55.6) | 16 (41.0) |
| Headache | 0 | 1 (16.7) | 0 | 1 (16.7) | 2 (33.3) | 4 (13.3) | 3 (33.3) | 7 (17.9) |
| Semen discoloration | 0 | 0 | 1 (16.7) | 0 | 3 (50.0) | 4 (13.3) | 0 | 4 (10.3) |
| Dizziness | 0 | 0 | 0 | 0 | 2 (33.3) | 2 (6.7) | 0 | 2 (5.1) |
| Nausea | 0 | 0 | 0 | 1 (16.7) | 1 (16.7) | 2 (6.7) | 0 | 2 (5.1) |
| Abdominal discomfort | 0 | 0 | 0 | 0 | 1 (16.7) | 1 (3.3) | 0 | 1 (2.6) |
| Abnormal sensation in eye | 0 | 0 | 0 | 0 | 1 (16.7) | 1 (3.3) | 0 | 1 (2.6) |
| Catheter site oedema | 0 | 0 | 0 | 0 | 1 (16.7) | 1 (3.3) | 1 (11.1) | 2 (5.1) |
| Catheter site pain | 0 | 0 | 0 | 0 | 1 (16.7) | 1 (3.3) | 0 | 1 (2.6) |
| Dyspepsia | 0 | 0 | 0 | 0 | 1 (16.7) | 1 (3.3) | 0 | 1 (2.6) |
| Flushing | 0 | 0 | 0 | 0 | 1 (16.7) | 1 (3.3) | 0 | 1 (2.6) |
| Infusion site discomfort | 0 | 0 | 0 | 0 | 1 (16.7) | 1 (3.3) | 0 | 1 (2.6) |
| Testicular embryonal carcinoma | 0 | 0 | 0 | 1 (16.7) | 0 | 1 (3.3) | 0 | 1 (2.6) |
| Testicular malignant teratoma | 0 | 0 | 0 | 1 (16.7) | 0 | 1 (3.3) | 0 | 1 (2.6) |
| Dry skin | 0 | 0 | 0 | 0 | 0 | 0 | 1 (11.1) | 1 (2.6) |
| Electrocardiogram T wave inversion | 0 | 0 | 0 | 0 | 0 | 0 | 1 (11.1) | 1 (2.6) |
| ^a^A subject with multiple AEs is counted only once in the “at least one AE” row. A subject with multiple AEs with the same preferred term is counted only once for that preferred term and treatment. Preferred terms are sorted in descending frequency, as reported in the pooled Cipargamin SAD group. AE, adverse event; IV, intravenous; SAD, single ascending dose. | | | | | | | | |

**Table S3. B. Incidences of adverse events of following IV administration of multiple ascending doses (MAD part) in healthy adults**

| **Adverse events (AEs)** | **Cipargamin**  **60 mg MAD** | **Cipargamin**  **120 mg MAD** | **Pooled cipargamin MAD** | **Pooled**  **placebo MAD** | **Total** |
| --- | --- | --- | --- | --- | --- |
|  | **N=6**  **n (%)** | **N=6**  **n (%)** | **N=12**  **n (%)** | **N=6**  **n (%)** | **N=18**  **n (%)** |
| Number of subjects with at least one AE^a^ | 4 (66.7) | 6 (100) | 10 (83.3) | 4 (66.7) | 14 (77.8) |
| Dizziness | 3 (50.0) | 4 (66.7) | 7 (58.3) | 1 (16.7) | 8 (44.4) |
| Headache | 2 (33.3) | 3 (50.0) | 5 (41.7) | 1 (16.7) | 6 (33.3) |
| Diarrhea | 0 | 2 (33.3) | 2 (16.7) | 2 (33.3) | 4 (22.2) |
| Fatigue | 0 | 2 (33.3) | 2 (16.7) | 1 (16.7) | 3 (16.7) |
| Abdominal discomfort | 0 | 1 (16.7) | 1 (8.3) | 1 (16.7) | 2 (11.1) |
| Alanine aminotransferase increased | 0 | 1 (16.7) | 1 (8.3) | 0 | 1 (5.6) |
| Arthralgia | 0 | 1 (16.7) | 1 (8.3) | 0 | 1 (5.6) |
| Aspartate aminotransferase increased | 0 | 1 (16.7) | 1 (8.3) | 0 | 1 (5.6) |
| Blood lactate dehydrogenase increased | 0 | 1 (16.7) | 1 (8.3) | 0 | 1 (5.6) |
| C-reactive protein increased | 0 | 1 (16.7) | 1 (8.3) | 0 | 1 (5.6) |
| Catheter site pain | 0 | 1 (16.7) | 1 (8.3) | 0 | 1 (5.6) |
| Dyspepsia | 0 | 1 (16.7) | 1 (8.3) | 0 | 1 (5.6) |
| Electrocardiogram abnormal | 1 (16.7) | 0 | 1 (8.3) | 0 | 1 (5.6) |
| Eructation | 1 (16.7) | 0 | 1 (8.3) | 0 | 1 (5.6) |
| Myalgia | 0 | 1 (16.7) | 1 (8.3) | 0 | 1 (5.6) |
| Nausea | 0 | 1 (16.7) | 1 (8.3) | 0 | 1 (5.6) |
| Neck pain | 0 | 1 (16.7) | 1 (8.3) | 0 | 1 (5.6) |
| Oral herpes | 1 (16.7) | 0 | 1 (8.3) | 0 | 1 (5.6) |
| Pruritus | 1 (16.7) | 0 | 1 (8.3) | 0 | 1 (5.6) |
| Vomiting | 0 | 1 (16.7) | 1 (8.3) | 0 | 1 (5.6) |
| Dysmenorrhea | 0 | 0 | 0 | 1 (16.7) | 1 (5.6) |
| Infusion related reaction | 0 | 0 | 0 | 1 (16.7) | 1 (5.6) |
| ^a^A subject with multiple AEs is counted only once in the “at least one AE” row. A subject with multiple AEs with the same preferred term is counted only once for that preferred term and treatment. Preferred terms are sorted in descending frequency, as reported in the Pooled cipargamin MAD group. AE, adverse event; IV, intravenous; MAD, multiple ascending dose. | | | | | |
